# Integrated transcriptomic and single cell analysis combined with artificial neural network identifies a robust gene signature for early discrimination of MASL and MASH

**DOI:** 10.3389/fimmu.2026.1813173

**Published:** 2026-04-20

**Authors:** Guo Wu Lin, Zhi Yuan Lin, Qi Yuan Su, Li Ye, Wei Ning Xu, Shun Qiang Nong, Ru Kai Wu, Wei Jie Zhou, Qian Fang Huang

**Affiliations:** 1 The Department of Laboratory, Baise People’s Hospital, Baise, Guangxi, China; 2 The Department of Endocrinology, Baise People’s Hospital, Baise, Guangxi, China; 3 Department of Gland Surgery, Affiliated Hospital of Youjiang Medical University for Nationalities, Key Laboratory of Tumor Molecular Pathology of Baise, Baise, Guangxi, China

**Keywords:** diagnostic biomarker, machine learning, MASH, MASLD, single cell RNA sequencing

## Abstract

**Background:**

Metabolic dysfunction-associated steatotic liver disease (MASLD) is the most prevalent chronic liver disease, ranging from simple steatosis (MASL) to metabolic dysfunction-associated steatohepatitis (MASH). However, reliable noninvasive strategies for accurately distinguishing MASL from MASH at an early stage remain limited. We therefore aimed to develop a robust molecular model to improve early identification of disease progression and subtype discrimination.

**Methods:**

Five datasets from the Gene Expression Omnibus were integrated as a training cohort comprising 149 MASL and 158 MASH samples, while another dataset GSE135251 served as validation cohort including 51 MASL and 155 MASH samples. Differential expression analysis and weighted gene co expression network analysis were conducted to identify gene modules. Overlapping genes were subjected to protein interaction network construction and topological ranking. Least absolute shrinkage and selection operator regression, support vector machine recursive feature elimination, and random forest algorithms were jointly applied to derive robust diagnostic candidates. An artificial neural network classifier was established based on the final gene set and evaluated in both cohorts. Immune cell composition was estimated using CIBERSORT. Single cell RNA sequencing data from GSE136103 were analyzed to determine cell type specific expression patterns. Quantitative real time PCR validation was conducted in 60 clinical liver tissue samples.

**Results:**

A total of 656 differentially expressed genes were identified between MASL and MASH. Network integration and machine learning intersection analysis consistently yielded six key genes: *MMP9, FABP5, TREM2, CTSD, UBD*, and *MAP2K1*. Five genes were upregulated in MASH, whereas MAP2K1 was downregulated. Individual genes demonstrated moderate diagnostic performance, with area under the curve values ranging from 0.692 to 0.822 in the training cohort. The artificial neural network model achieved an area under the curve of 0.893 (95% CI 0.854 to 0.925) in the validation cohort. Immune infiltration analysis revealed increased monocytes, M0 and M1 macrophages, and activated dendritic cells in MASH. Single cell analysis localized key genes predominantly to myeloid populations, and quantitative PCR confirmed consistent differential expression in clinical samples.

**Conclusion:**

This study establishes a multicohort machine learning-based gene signature with high diagnostic accuracy for distinguishing MASL from MASH and provides insight into immune metabolic mechanisms underlying disease progression.

## 1 Introduction

Metabolic dysfunction-associated steatotic liver disease (MASLD), formerly nonalcoholic fatty liver disease (NAFLD), is the most prevalent chronic liver disease worldwide. A recent meta-analysis estimated that nearly 30 percent of the global adult population is affected, with the prevalence exceeding 50 percent among individuals with obesity (1). Updated epidemiological data further indicate that MASL accounts for approximately 78 percent of all chronic liver diseases, corresponding to an estimated 1.235 billion cases globally (2). Of these patients, nearly one quarter of patients progress to nonalcoholic steatohepatitis (MASH), a more aggressive phenotype characterized by hepatocellular injury and inflammation that may culminate in fibrosis, cirrhosis, and hepatocellular carcinoma (HCC) (3). MASH has become a leading indication for liver transplantation in many developed countries, and mortality related to MASL and MASH continues to rise (4). As obesity and type 2 diabetes remain highly prevalent, the incidence and clinical burden of MASL and MASH are expected to escalate further.

The transition from MASL to MASH reflects a complex interplay of metabolic, inflammatory, and cellular stress pathways. The widely accepted multiple hit hypothesis proposes that insulin resistance driven by obesity, excessive lipid accumulation within hepatocytes, oxidative stress, and the release of proinflammatory mediators act in concert to promote hepatocellular damage and progressive fibrosis in susceptible individuals (5, 6). Experimental and clinical observations consistently show that only a subset of patients with simple steatosis develop significant necroinflammation and fibrotic remodeling, underscoring the heterogeneity of disease progression (6, 7). Despite this growing understanding, effective pharmacological therapies remain limited, and lifestyle modification continues to be the cornerstone of management. Diagnostic strategies face comparable challenges. Liver biopsy remains the reference standard for diagnosing MASH, yet its invasive nature, limited patient acceptance, high cost, sampling variability, and potential complications such as bleeding and infection restrict widespread application (8, 9). Imaging modalities, including elastography and magnetic resonance imaging proton density fat fraction, can quantify hepatic steatosis and fibrosis, but their expense and limited accessibility preclude large scale population screening (10). Serum biomarkers offer practical advantages; however, reported candidates show variable performance and insufficient specificity, making accurate discrimination between MASL and MASH difficult in routine practice (11). Most patients remain asymptomatic in early stages (12), and conventional liver function tests or composite risk scores lack the precision required to identify early MASH. This diagnostic gap raises a fundamental question: can molecular signatures derived from high dimensional data improve early detection and risk stratification?

To address this need, recent investigations have applied multi omics integration and machine learning approaches to identify candidate biomarkers (13). By combining transcriptomic and metabolomic profiles with protein interaction networks and algorithms such as least absolute shrinkage and selection operator, support vector machines, and random forest models, several groups have constructed diagnostic signatures with encouraging performance (13). For instance, Hasin Brumshtein and colleagues integrated liver transcriptomic data from 12 independent cohorts comprising 812 samples, identified 130 MASH associated genes, and derived a 19 gene signature through forward search that demonstrated robust discrimination in both discovery and validation sets (3). Nevertheless, many studies remain constrained by modest sample sizes, limited data sources, and insufficient external validation. One machine learning analysis acknowledged that its relatively small transcriptomic dataset and data merging procedures might have resulted in information loss, emphasizing the need for larger and multicenter cohorts (14). In parallel, insight into the single cell molecular landscape of MASL and MASH is still evolving. Earlier single cell studies have largely relied on animal models, and cell type specific transcriptional alterations in human liver tissue have not been comprehensively characterized (15). These limitations inevitably affect the reproducibility and generalizability of proposed biomarkers.

Building upon these considerations, the present study implemented a multicenter data integration framework combined with layered analytical strategies. Multiple publicly available transcriptomic cohorts were harmonized to generate a large training dataset after batch effect correction. Weighted gene co expression network analysis and differential expression analysis were then applied to identify modules and genes closely associated with disease severity. A protein protein interaction network was constructed, and three complementary machine learning methods, including least absolute shrinkage and selection operator regression, support vector machine recursive feature elimination, and random forest, were jointly employed to refine key genes. Based on these candidates, an artificial neural network model was developed to enhance the discriminatory performance between MASL and MASH. To further elucidate biological relevance, the selected genes were mapped onto single cell transcriptomic datasets to determine their cell type specific expression patterns within the human liver. Immune infiltration analysis and validation in clinical samples were subsequently performed to strengthen mechanistic interpretation. By integrating multicohort bulk transcriptomic data, single cell resolution analysis, diverse machine learning algorithms, and experimental validation, this strategy enhances the robustness of biomarker discovery while providing a more comprehensive view of the molecular events underlying MASL and MASH progression. Such an approach may facilitate earlier molecular diagnosis and improve the interpretability and clinical applicability of predictive models in this increasingly prevalent liver disease spectrum.

## 2 Methods

### 2.1 Assembly of transcriptomic cohorts and study design

Transcriptomic datasets associated with nonalcoholic fatty liver disease were collected from the Gene Expression Omnibus repository (https://www.ncbi.nlm.nih.gov/geo/). Cohorts were eligible if diagnoses of nonalcoholic fatty liver or nonalcoholic steatohepatitis were confirmed by histopathological evaluation, gene expression matrices were available in either raw or normalized form, and sample numbers were sufficient for downstream statistical analyses. Six independent cohorts fulfilled these requirements, including GSE48452, GSE66676, GSE89632, GSE130970, GSE135251, and GSE167523, representing patient populations from Germany, the United States, Canada, the United Kingdom, and Japan. Five datasets were integrated to establish the training cohort, yielding 149 MASL and 158 MASH samples, whereas GSE135251, which contained the largest number of cases, was reserved as an external validation cohort comprising 51 MASL and 155 MASH samples. This analytical framework enabled cross population integration while preserving an independent dataset for model validation. Additionally, the single cell RNA sequencing dataset GSE136103 was incorporated to examine the distribution of candidate gene expression across hepatic cell types derived from normal and cirrhotic liver tissues, thereby providing cell level support for biological relevance. The dataset was used to explore cell-type-specific expression patterns rather than disease stage comparisons.

### 2.2 Data processing and normalization

Expression matrices corresponding to the five training cohorts, including GSE48452, GSE66676, GSE89632, GSE130970, and GSE167523, were obtained from the GEO repository. Probe level identifiers were converted to gene symbols using platform specific annotation files, and mean expression values were calculated when multiple probes mapped to the same gene. Genes shared across all datasets were retained to ensure consistency in downstream analyses. Following gene filtering, expression profiles derived from individual cohorts were integrated into a unified matrix, with batch labels assigned according to dataset origin. To minimize technical variability introduced by differences in experimental platforms and cohort sources, batch effects were adjusted using the ComBat algorithm implemented in the sva R package (https://bioconductor.org/packages/release/bioc/html/sva.html). For datasets providing continuous normalized expression values, logarithmic transformation using log2(x+1) was applied prior to batch correction. Data distribution characteristics and global variance structure were subsequently evaluated through principal component analysis and boxplot visualization using the ggplot2 (https://cran.r-project.org/package=ggplot2) and ggpubr (https://cran.r-project.org/package=ggpubr) packages in R. To further evaluate the effectiveness of batch correction, both distributional and dimensionality reduction approaches were applied. Boxplot visualization was used to assess the consistency of gene expression distributions across datasets, while principal component analysis was performed to examine sample clustering patterns before and after correction. Prior to batch correction, samples were clearly separated according to dataset origin, indicating substantial batch effects. After correction, expression distributions became comparable across datasets, and PCA demonstrated effective integration with reduced batch-driven clustering, supporting the reliability of downstream analyses.

### 2.3 Identification of differentially expressed genes and co-expression network analysis

Differential transcriptional profiles between MASL and MASH samples were examined within the integrated training cohort using linear modeling implemented in the limma framework (https://bioconductor.org/packages/release/bioc/html/limma.html). Group comparisons were specified through predefined contrasts distinguishing MASH from MASL, and genes meeting the criteria of an adjusted P value below 0.05 together with an absolute log2 fold change exceeding two were defined as significantly altered. A stringent threshold (|log_2_FC| > 2) was applied to identify robust differentially expressed genes, aiming to reduce potential noise and highlight genes with more pronounced biological relevance. Global expression differences among these genes were visualized through volcano plots and hierarchical heatmaps generated with ggplot2 (https://cran.r-project.org/package=ggplot2) and pheatmap (https://cran.r-project.org/package=pheatmap). The soft-thresholding power (β) was determined based on the scale-free topology criterion. Outlier samples were removed using hierarchical clustering with a cut height of 20,000. To characterize coordinated gene expression patterns associated with disease status, weighted gene co-expression network analysis was performed using the WGCNA package (https://cran.r-project.org/package=WGCNA). Genes exhibiting minimal variance were excluded prior to network construction, and quality control procedures were applied to remove aberrant samples. An adjacency matrix was established using a selected soft thresholding power and subsequently converted into a topological overlap matrix. Gene clustering was carried out based on topological overlap dissimilarity, and dynamic tree cutting was employed to define gene modules with a minimum size of fifty genes, followed by merging of highly similar modules. Module eigengenes were then correlated with clinical phenotypes to identify co-expression modules most strongly associated with MASL and MASH status.

### 2.4 Construction of interaction networks and identification of key genes

Genes shared between the differentially expressed gene set and disease associated co expression modules were selected for interaction network analysis. Information on gene level interactions was retrieved from the STRING database (https://string-db.org/). The interaction network was subsequently visualized using Cytoscape software version 3.10.1. To prioritize central genes within the network, the cytoHubba plugin was applied, and gene importance was evaluated using four topological ranking approaches, namely Degree, EPC, MCC, and MNC, as summarized in **Supplementary Table 1**. For each algorithm, the top twenty ranked genes were extracted, and genes consistently identified across all methods were defined as key candidates for downstream analyses. Overlapping gene sets were visualized using Venn diagrams generated in R with the ggvenn package (https://cran.r-project.org/package=ggvenn).

### 2.5 Machine learning driven selection of key genes

To further narrow the set of disease relevant genes, three complementary machine learning strategies were applied to the key genes obtained from the protein interaction network. Least absolute shrinkage and selection operator regression was implemented using the glmnet package (https://cran.r-project.org/package=glmnet), where a binomial logistic regression framework was adopted and ten fold cross validation was performed to determine the optimal penalty parameter. Genes retaining non zero coefficients at the selected parameter value were preserved as candidate features. LASSO was performed using the glmnet package with a binomial model and alpha set to 1. Ten-fold cross-validation was conducted using the cv.glmnet function, and the optimal penalty parameter was determined based on lambda.min, corresponding to the minimum cross-validation error. In parallel, support vector machine based recursive feature elimination was conducted with the e1071 package (https://cran.r-project.org/package=e1071) together with an in house R workflow (https://zenodo.org/records/19254731), in which samples were classified into MASL and MASH groups and feature ranking was achieved through iterative elimination combined with ten fold cross validation, retaining genes with the highest importance scores. Support vector machine recursive feature elimination was implemented using a linear kernel, and feature selection was carried out through iterative elimination combined with ten-fold cross-validation to identify the optimal subset of genes. Random forest analysis was subsequently carried out using the randomForest package (https://cran.r-project.org/package=randomForest), generating five hundred decision trees, with model performance optimized by minimizing classification error. Gene importance was quantified using the mean decrease Gini metric, and genes exceeding the predefined threshold were selected. Random forest analysis was conducted with an initial setting of 500 trees, and the optimal number of trees was determined by minimizing the out-of-bag error. The mtry parameter was set to the default value, corresponding to the square root of the number of input variables. Genes with a mean decrease Gini value greater than 8 were considered important features. Genes consistently identified across all three approaches were defined as the final set of key genes for subsequent analyses.

### 2.6 Expression profiling and diagnostic assessment of key genes

Expression measurements of the final set of key genes were obtained from both the integrated training cohort and the independent validation dataset GSE135251. Comparative analyses of gene expression between MASL and MASH samples were visualized using boxplots generated with ggpubr and ggplot2, and statistical significance was evaluated using the Wilcoxon rank sum test. The diagnostic utility of each gene was assessed through receiver operating characteristic analysis, with calculation of the area under the curve performed using the pROC package (https://cran.r-project.org/package=pROC). All evaluations were conducted independently in the training and validation cohorts to ensure consistency of diagnostic performance.

### 2.7 Construction of an artificial neural network based on key genes

An artificial neural network classifier was established using expression profiles of the final key genes derived from the integrated training cohort. Sample categories corresponding to MASL and MASH were encoded using a one hot representation. Model training was performed with the neuralnet package (https://cran.r-project.org/package=neuralnet) employing a single hidden layer composed of four neurons and a maximum of one million training iterations, with a fixed random seed to ensure reproducibility. Network architecture and connection weights were visualized using NeuralNetTools (https://cran.r-project.org/package=NeuralNetTools). Class probabilities were generated through the compute function, and predicted class labels were assigned based on the node with the highest output value. Model discrimination was evaluated using receiver operating characteristic analysis, and the area under the curve was calculated with the pROC package, with bootstrap based confidence intervals applied where appropriate. All data preprocessing steps, including probe annotation and gene level aggregation, followed the procedures described above using limma, and the same modeling workflow was subsequently applied to the independent validation cohort GSE135251 for external assessment. A single hidden layer with four neurons was selected to balance model complexity and generalizability, given the limited number of input features. Extensive hyperparameter tuning was not performed, as the primary objective was to construct a stable and interpretable model. To reduce the risk of overfitting, feature selection was conducted using multiple machine learning approaches with cross-validation, and model performance was further evaluated in an independent external validation cohort.

### 2.8 Drug association analysis and construction of a ceRNA regulatory network

To explore potential therapeutic relevance and regulatory mechanisms associated with the key genes, two complementary downstream analyses were carried out. Drug association analysis was performed using the DSigDB_All_detailed dataset obtained from the DSigDB resource (https://dsigdb.tanlab.org/), from which drug to gene mappings were extracted as TERM2GENE entries. Enrichment analysis was conducted using the enricher function implemented in clusterProfiler (https://bioconductor.org/packages/clusterProfiler), with significance thresholds defined as nominal P values below 0.05 and adjusted P values below 0.05, as summarized in **Supplementary Table 2**. Enriched drug associations were visualized using barplot and dotplot functions provided by enrichplot and further formatted with ggplot2. In parallel, a competing endogenous RNA regulatory network was constructed to investigate post transcriptional regulation. Interactions between key genes and microRNAs were predicted by integrating results from miRanda, miRDB, TargetScan, and miRWalk, retaining only microRNAs supported by all four databases. Long non coding RNA to microRNA interactions were subsequently obtained from spongeScan, and shared nodes were integrated with gene microRNA pairs to assemble the ceRNA network. Network edge and node information was exported and visualized using Cytoscape software version 3.10.1.

### 2.9 Estimation of immune cell composition and gene associations

Immune cell composition within the training cohort was estimated using the CIBERSORT algorithm (https://cibersort.stanford.edu/) in conjunction with the LM22 reference signature and one thousand permutations, retaining only samples that met the predefined significance criterion of P values below 0.05. Spearman correlation analysis was performed to assess associations between gene expression and immune cell fractions. Estimated immune cell proportions were visualized through stacked bar charts and boxplots generated using ggplot2 and ggpubr. Associations between key gene expression levels and immune cell fractions were evaluated using Spearman correlation analysis, and gene immune interaction patterns were subsequently visualized with the linkET package (https://github.com/Hy4m/linkET).

### 2.10 Single cell transcriptomic analysis and cell type annotation

The single cell transcriptomic dataset GSE136103 was analyzed using the Seurat framework (https://cran.r-project.org/package=Seurat). Quality control procedures were applied to exclude low-quality cells, defined as those with low gene feature counts or elevated mitochondrial transcript proportions (>15%), prior to downstream analysis. Following filtering, expression data were normalized and scaled, and highly variable genes were identified for subsequent analyses. Dimensionality reduction was carried out using principal component analysis, followed by t distributed stochastic neighbor embedding for visualization. Cells were clustered based on transcriptional similarity, and marker genes were identified for each cluster. Cell type annotation was performed with SingleR (https://bioconductor.org/packages/SingleR) using reference transcriptomic datasets. To characterize the cellular distribution of key genes, violin plots and dot plots were generated to depict expression patterns across annotated cell populations. In addition, differential expression analyses comparing MASL and MASH samples were conducted within individual cell types. Visualization of single cell results was performed using Seurat in combination with ggplot2 and ggpubr. Differential expression analyses within each annotated cell type were performed independently using the Wilcoxon test implemented in Seurat. Given the cell-type-specific analytical framework, comparisons were conducted within predefined cell populations.

### 2.11 Clinical tissue collection and quantitative real time PCR validation

Pathological liver tissue specimens were retrospectively identified and collected from the electronic medical record system of Baise People’s Hospital, with all samples obtained from the institutional specimen repository, yielding a total of sixty cases. Ethical approval for this study was granted by the Ethics Committee of Baise People’s Hospital. Total RNA was isolated from tissue samples using TRIzol reagent from TAKARA according to the manufacturer’s protocol. Transcript levels of *MMP9, FABP5, TREM2, CTSD, UBD*, and *MAP2K1* were quantified by quantitative real time polymerase chain reaction following previously reported procedures, with β actin serving as the internal reference for normalization. All amplification reactions were performed in technical triplicate on a Bio Rad CFX96 real time PCR system, and relative expression levels were calculated using the comparative Ct approach. Relative gene expression levels were calculated using the 2^−ΔΔCt method. ΔCt was defined as the difference between the target gene and the internal control gene (β-actin). ΔΔCt was calculated by subtracting the mean ΔCt of the control group from that of each sample, and relative expression levels were expressed as 2^−ΔΔCt. RNA quality and purity were assessed prior to qPCR analysis, and only samples meeting standard quality criteria were included. β-actin was selected as the reference gene due to its stable expression across samples and its common use as an internal control in similar studies. Primer sequences used for amplification are provided in **Table 1**.

**Table 1:** Primer sequence.

Gene	Forward primer sequence (5’ -> 3’)	Reverse primer sequence (5’ -> 3’)
*MMP9*	TGTACCGCTATGGTTACACTCG	GGCAGGGACAGTTGCTTCT
*FABP5*	TGAAGGAGCTAGGAGTGGGAA	TGCACCATCTGTAAAGTTGCAG
*TREM2*	CCTGGGAGATACAGACAATGC	GGTCCTGGAACACAAGGAGG
*CTSD*	TGCTCAAGAACTACATGGACGC	CGAAGACGACTGTGAAGCACT
*UBD*	CCGTTCCGAGGAATGGGATTT	GCCATAAGATGAGAGGCTTCTCC
*MAP2K1*	CAATGGCGGTGTGGTGTTC	GATTGCGGGTTTGATCTCCAG
ACTIN	AAGGAGCCCCACGAGAAAAAT	ACCGAACTTGCATTGATTCCAG

### 2.12 Statistical analysis

All statistical analyses were conducted using R software version 4.3.0. Continuous variables are presented as medians with interquartile ranges unless otherwise indicated. Comparisons between two groups were performed using the Wilcoxon rank sum test for non normally distributed data. Differential expression analysis was carried out using linear modeling implemented in the limma package, with false discovery rate control applied through the Benjamini Hochberg procedure. Associations between gene expression levels and immune cell proportions were evaluated using Spearman correlation analysis. Diagnostic performance was assessed by receiver operating characteristic curve analysis, and the area under the curve was calculated using the pROC package, with bootstrap resampling applied to estimate confidence intervals when appropriate. In machine learning analyses, ten fold cross validation was used to limit overfitting and to evaluate model stability, and feature importance and performance metrics were derived according to the corresponding algorithms. For single cell transcriptomic analyses, differential gene expression between MASL and MASH samples within each annotated cell type was assessed using the Wilcoxon test implemented in Seurat. Relative gene expression levels obtained from quantitative real time PCR experiments were compared between groups using the Wilcoxon rank sum test. All statistical tests were two sided, and P values below 0.05 were considered statistically significant unless otherwise specified.

## 3 Results

### 3.1 Integration of transcriptomic datasets and evaluation of batch effects

**Figure 1:**
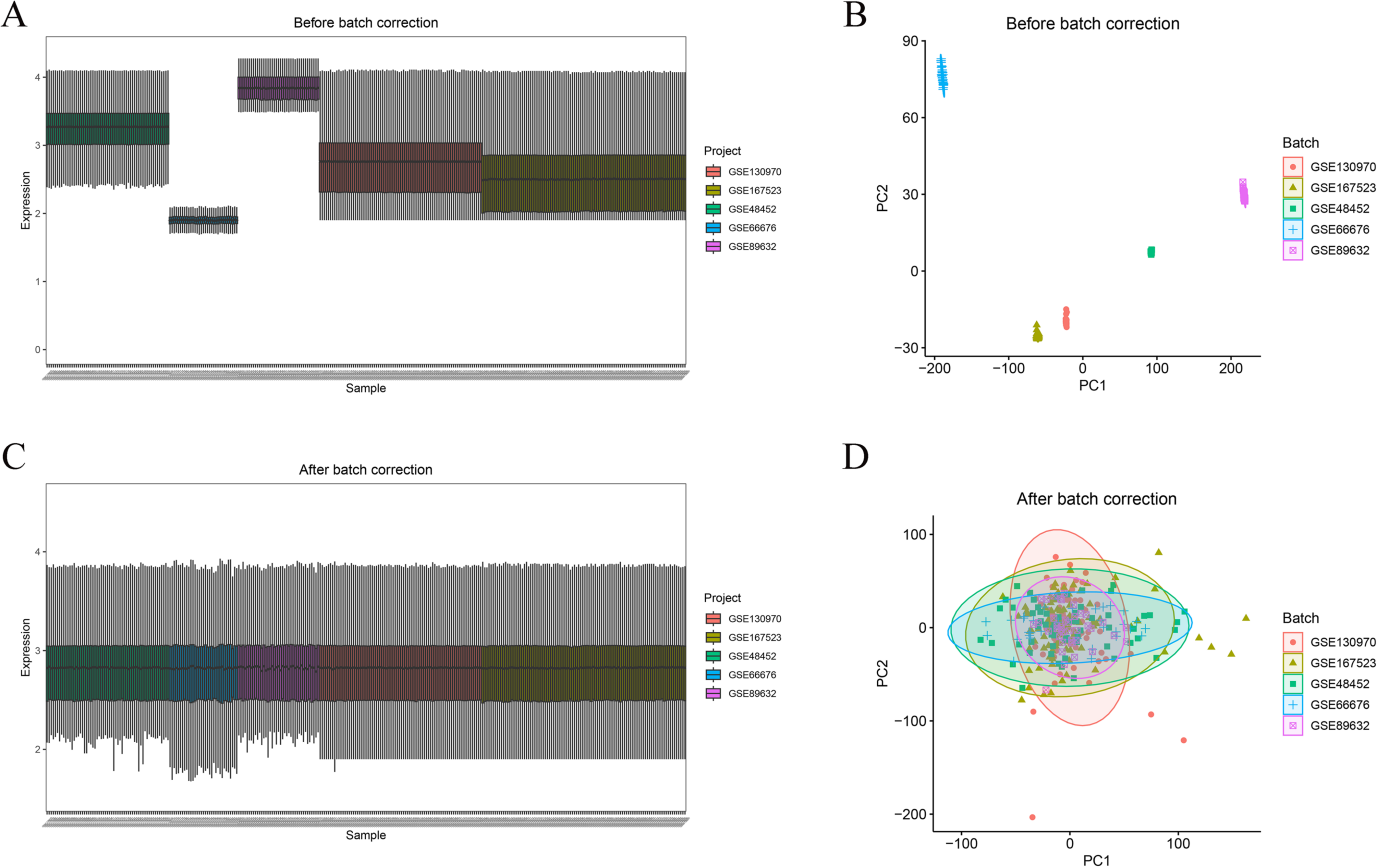
Batch correction and data integration. **(A)** Boxplot showing gene expression distributions before batch correction. **(B)** PCA plot showing strong dataset-specific clustering prior to correction. **(C)** Boxplot after batch correction showing harmonized distributions. **(D)** PCA after correction demonstrating effective integration of samples across cohorts. PCA, Principal component analysis.

Five independent GEO datasets were combined to generate the training cohort, comprising 149 MASL samples and 158 MASH samples. A total of 14,533 overlapping genes were retained after dataset intersection prior to batch correction. Prior to batch effect adjustment, marked variability in gene expression distributions was observed across datasets, with both boxplot visualization and principal component analysis indicating that sample clustering was largely driven by dataset origin rather than disease status, as shown in **Figures 1A, B**. Following correction using the ComBat approach, expression profiles across cohorts became highly comparable, and principal component analysis demonstrated effective integration of samples without evident batch associated separation, as illustrated in **Figures 1C, D**. These findings indicate successful mitigation of batch effects and support the reliability of the integrated dataset for subsequent analyses.

### 3.2 Differential transcriptional changes and identification of key gene sets

**Figure 2:**
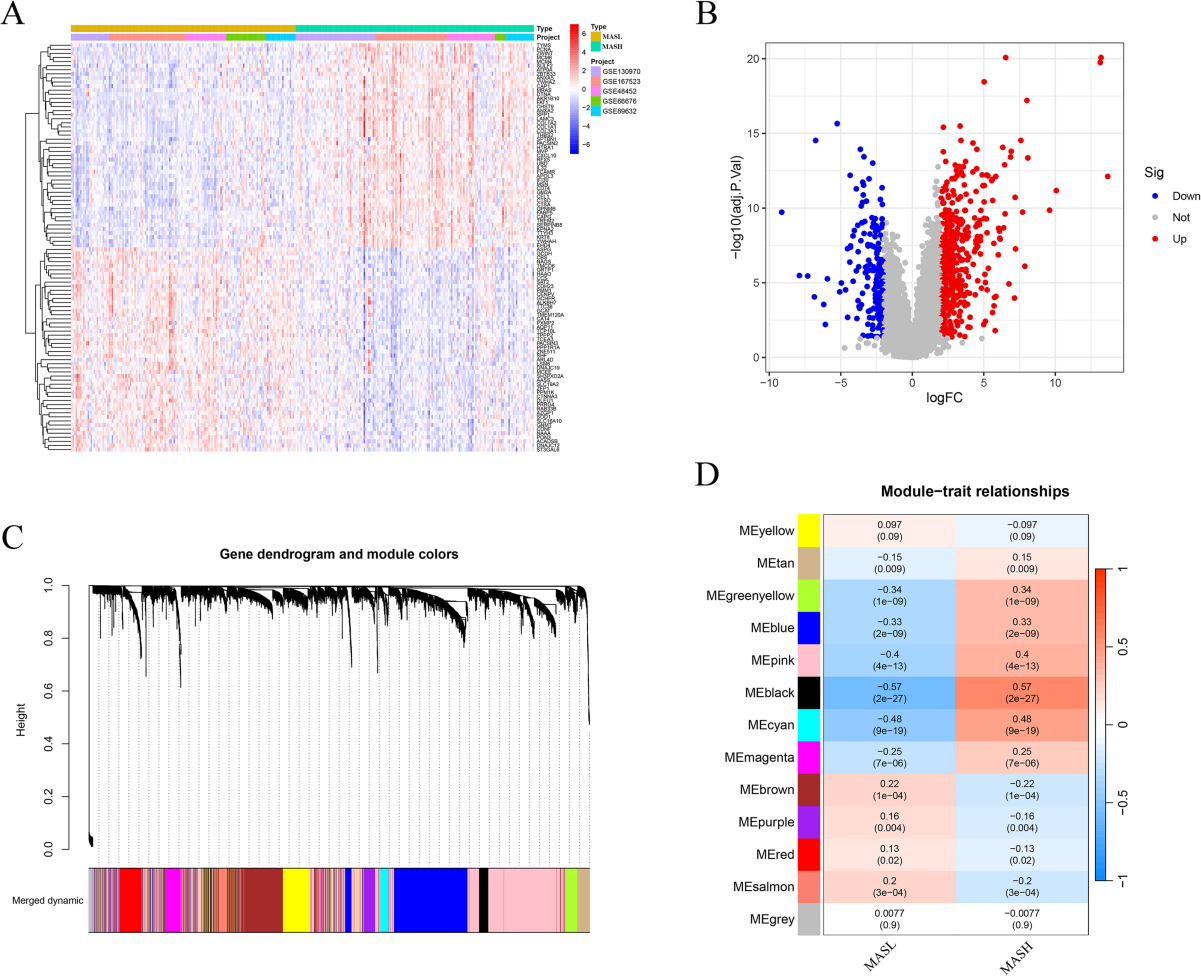
Differential expression and WGCNA analysis. **(A)** Heatmap of DEGs between MASL and MASH. **(B)** Volcano plot of DEGs (red: upregulated, blue: downregulated). **(C)** Gene dendrogram with module colors identified by WGCNA. **(D)** Heatmap of module–trait correlations with MASL and MASH. DEGs, differentially expressed genes; MASL, non-alcoholic fatty liver; MASH, non-alcoholic steatohepatitis; WGCNA, weighted gene co-expression network analysis.

**Figure 3:**
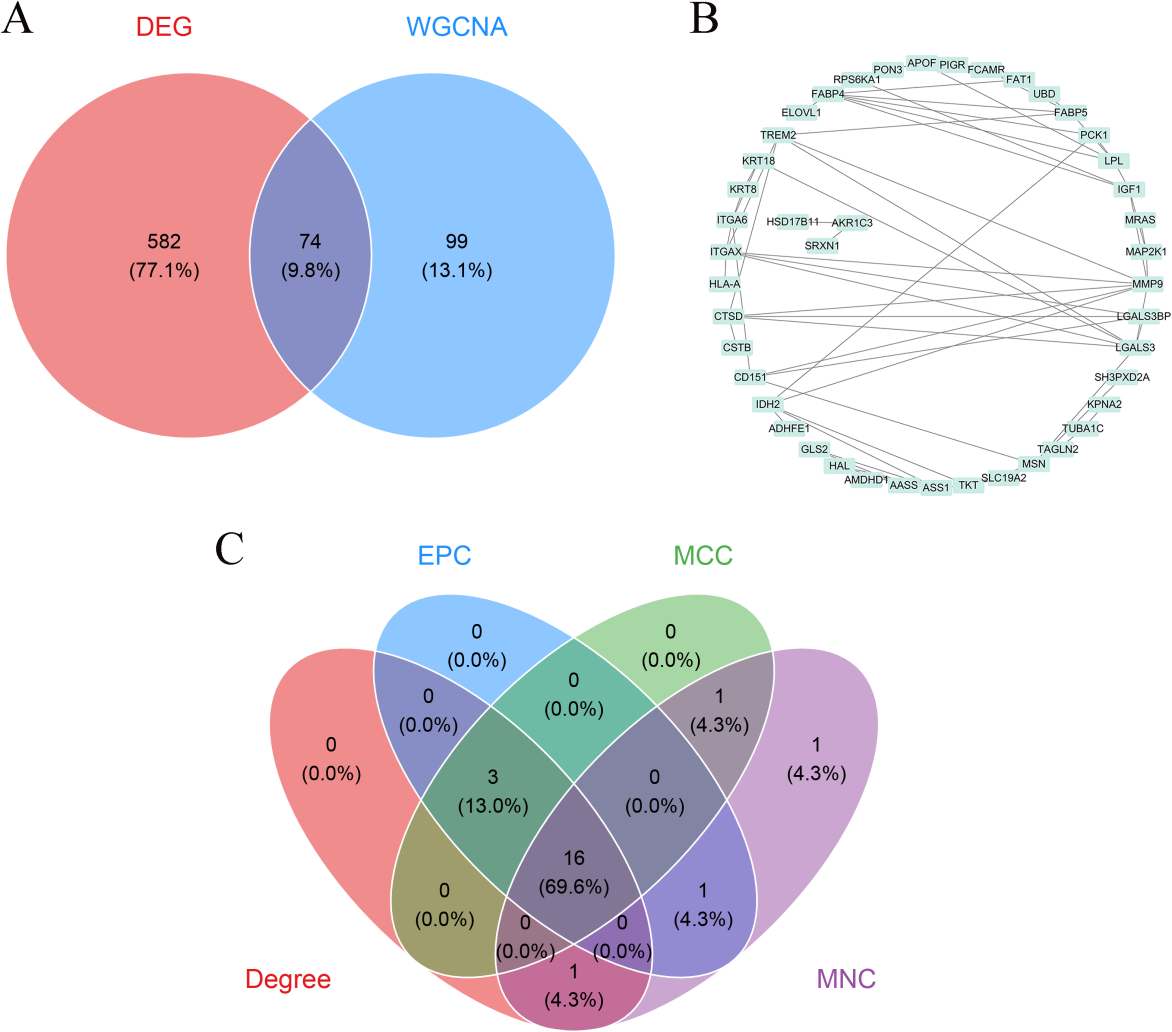
Identification of key genes. **(A)** Venn diagram showing overlap between DEGs and WGCNA modules. **(B)** PPI network of overlapping genes constructed from STRING(https://string-db.org/). **(C)** Venn diagram of key genes identified by four cytoHubba algorithms. DEGs, differentially expressed genes; WGCNA, weighted gene co-expression network analysis; PPI, protein–protein interaction.

Comparative analysis of transcriptomic profiles between MASL and MASH samples identified 656 differentially expressed genes, comprising 477 genes with increased expression and 179 genes with reduced expression in MASH (**Supplementary Table 4**). Unsupervised hierarchical clustering based on these genes clearly distinguished MASL from MASH samples, as illustrated in the heatmap shown in **Figure 2A**, while the corresponding volcano plot summarized the extent and direction of expression changes across the transcriptome in **Figure 2B**. To characterize coordinated expression patterns linked to disease status, weighted gene co expression network analysis was conducted, leading to the detection of multiple gene modules. Among these modules, the black module exhibited the strongest association with MASH, with a Pearson correlation coefficient of 0.57 and an adjusted P value below 0.001, as shown in **Figures 2C, D**, indicating a potential role in disease progression from MASL to MASH. To refine disease relevant candidates, genes shared between the differentially expressed gene set and MASH associated modules were identified, yielding 74 overlapping genes. These genes were subsequently used to construct a protein interaction network based on STRING data and visualized using Cytoscape, as shown in **Figure 3B**. Key gene prioritization was performed using the cytoHubba plugin with four topological ranking methods, including Degree, EPC, MCC, and MNC, and intersection analysis revealed 16 genes that consistently ranked among the top twenty across all algorithms, as depicted in **Figure 3C**. These genes were defined as key genes and selected for subsequent analyses.

### 3.3 Machine learning driven identification and validation of key diagnostic genes

**Figure 4:**
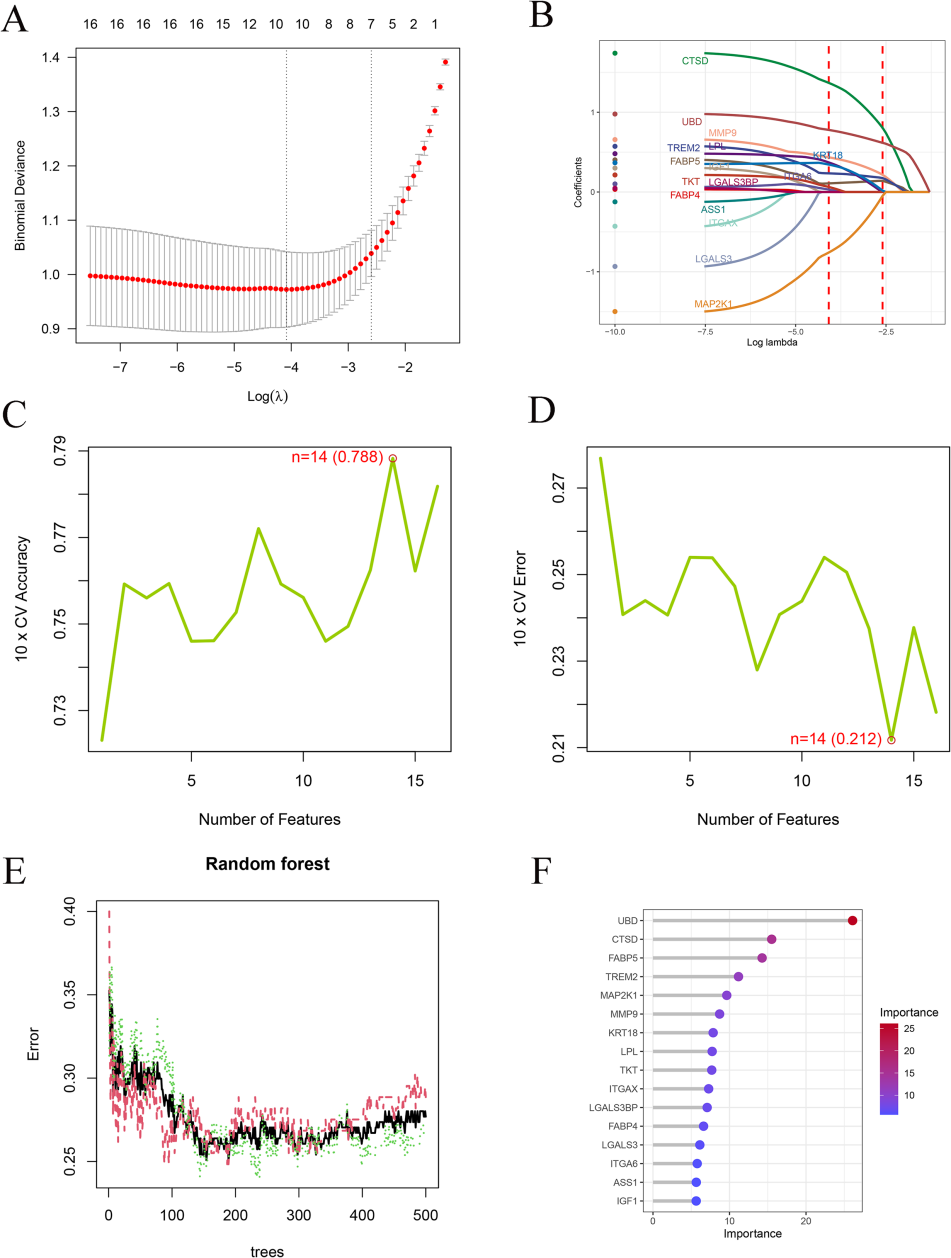
Machine learning-based feature selection. **(A, B)** LASSO regression analysis identifying genes with non-zero coefficients. **(C, D)** SVM-RFE model showing optimal feature number with maximum accuracy and minimum error. **(E, F)** Random Forest analysis showing error rates across trees and importance ranking of genes. LASSO, least absolute shrinkage and selection operator; SVM-RFE, support vector machine–recursive feature elimination.

**Figure 5:**
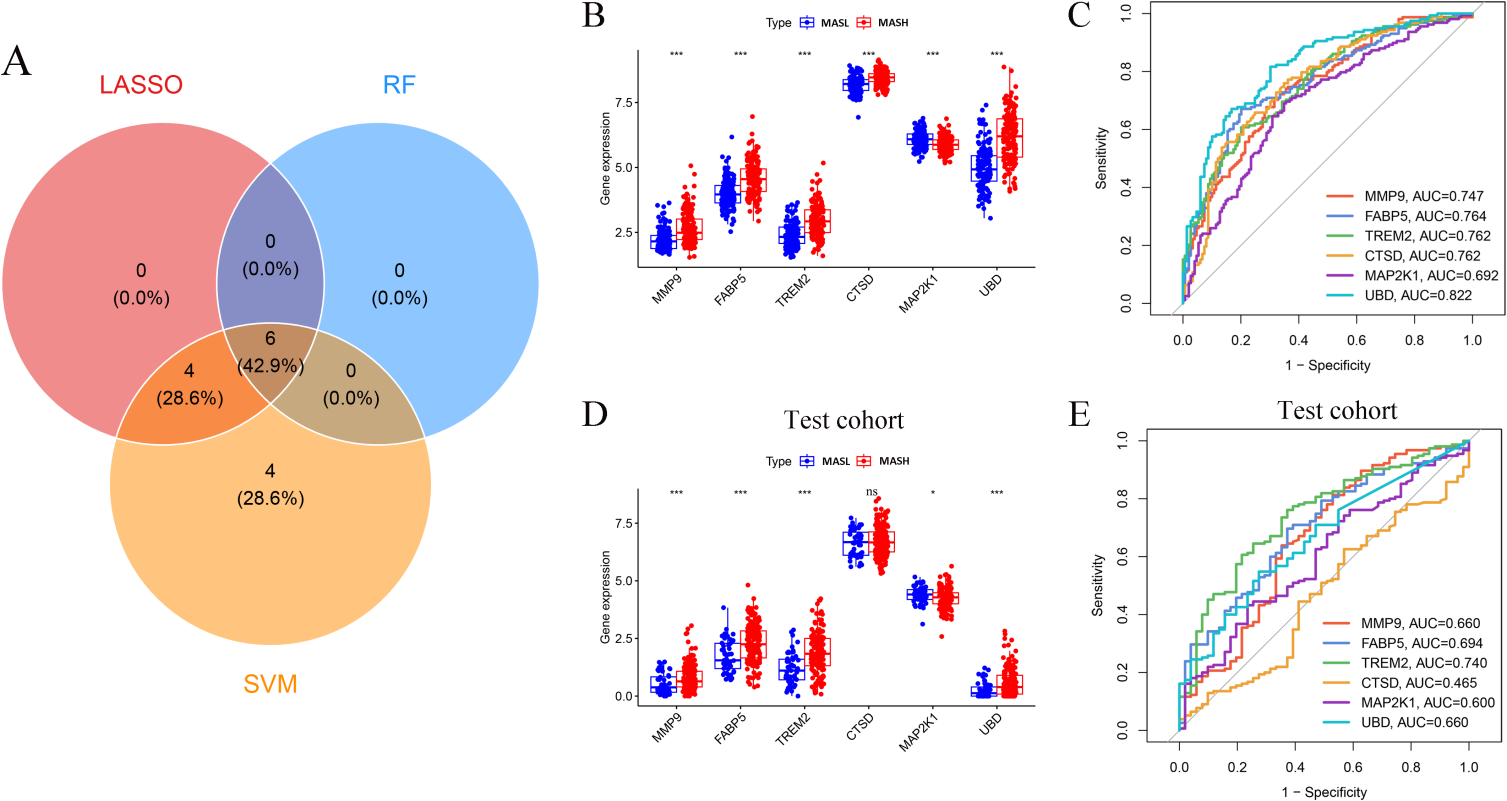
Validation of key genes. **(A)** Venn diagram showing intersection of LASSO, SVM-RFE, and RF selected genes. **(B)** Expression of six key genes in the training cohort. **(C)** ROC curves for key genes in the training cohort. **(D)** Expression validation in the independent cohort (GSE135251). **(E)** ROC curves for key genes in the independent cohort. LASSO, least absolute shrinkage and selection operator; SVM-RFE, support vector machine–recursive feature elimination; RF, random forest; ROC, receiver operating characteristic. * P < 0.05; *** P < 0.001.

To derive a robust diagnostic gene signature for MASH, three independent machine learning approaches were employed for feature selection. Least absolute shrinkage and selection operator regression retained ten genes with non zero coefficients at the optimal regularization parameter, as illustrated in **Figures 4A, B**. Support vector machine based recursive feature elimination achieved maximal cross validation accuracy when fourteen genes were preserved, as shown in **Figures 4C, D**. In parallel, random forest modeling based on five hundred decision trees ranked gene importance and identified six genes with strong discriminatory capacity, as presented in **Figures 4E, F**. Intersection analysis across the three algorithms consistently yielded six overlapping genes, namely *MMP9, FABP5, TREM2, CTSD, MAP2K1*, and *UBD*, which were defined as key diagnostic genes for further evaluation, as depicted in **Figure 5A**. Expression profiling revealed that *MMP9, FABP5, TREM2, CTSD*, and *UBD* exhibited significantly higher expression in MASH samples than in MASL samples within the training cohort, whereas *MAP2K1* showed the opposite trend, as shown in **Figure 5B**. Receiver operating characteristic analysis demonstrated moderate to favorable diagnostic performance for individual genes, with area under the curve values ranging from 0.692 to 0.822 in the training cohort, as illustrated in **Figure 5C**. Independent validation in the GSE135251 cohort confirmed increased expression of *MMP9, FABP5*, *TREM2*, and *UBD* and reduced expression of *MAP2K1*, while *CTSD* did not reach statistical significance, as shown in **Figure 5D**. Consistent with these findings, receiver operating characteristic analysis in the validation cohort yielded area under the curve values ranging from 0.465 to 0.740, as presented in **Figure 5E**. Collectively, these results demonstrate that the six gene signature displays reproducible expression patterns across cohorts and retains diagnostic potential for distinguishing MASH from MASL.

### 3.4 Performance of an artificial neural network based diagnostic model

**Figure 6:**
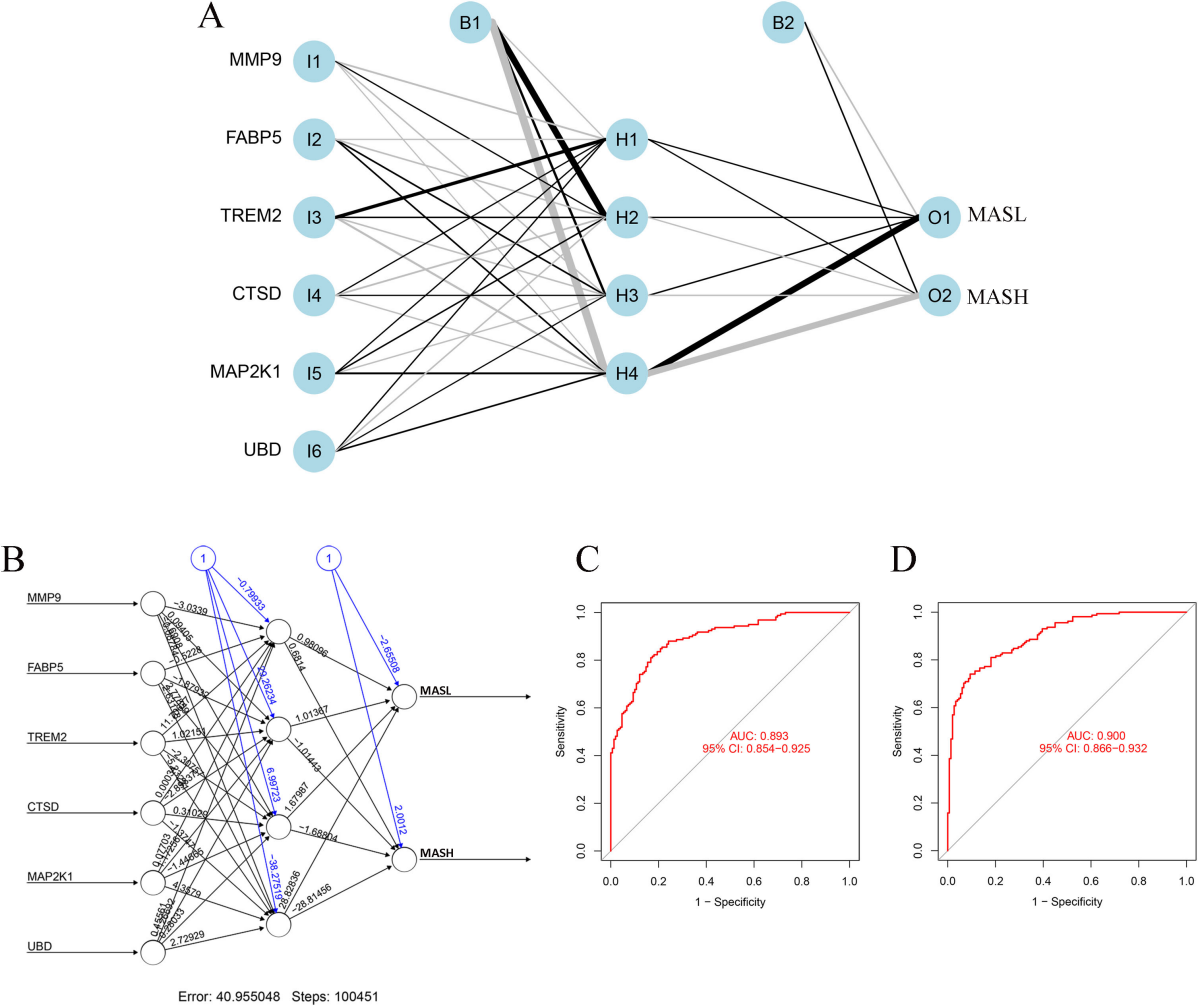
Artificial Neural Network (ANN) model. **(A, B)** Structure and weight visualization of the ANN model. **(C, D)** ROC curve the validation **(C)** and training **(D)** demonstrating diagnostic performance of the ANN model. ANN, artificial neural network; ROC, receiver operating characteristic.

An artificial neural network model was constructed using the six key diagnostic genes to improve classification performance between MASL and MASH samples. The model architecture consisted of six input units, a single hidden layer containing four neurons, and two output units representing the two disease states, as shown in **Figure 6A**. Network weights and connectivity patterns are illustrated in **Figure 6B**. As shown in **Figures 6C, D**, the ANN model demonstrated strong discriminatory performance, with an area under the receiver operating characteristic curve (AUC) of 0.893 (95% CI: 0.854–0.925) in the validation cohort and 0.900 (95% CI: 0.866–0.932) in the training cohort. This performance exceeded that of classifiers based on individual genes.

### 3.5 Drug associated signatures and ceRNA regulatory network features

**Figure 7:**
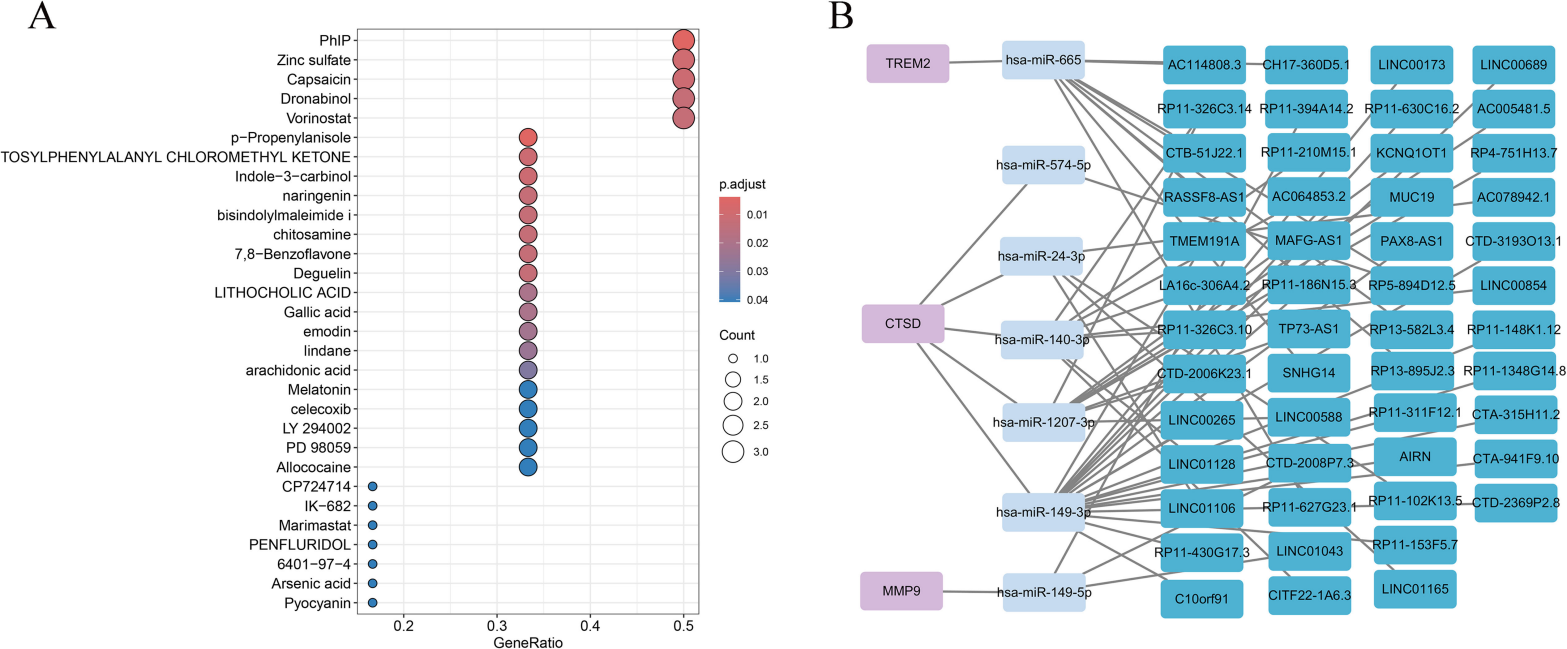
Drug enrichment and ceRNA network. **(A)** Bubble plot of enriched drugs associated with the six key genes. **(B)** ceRNA network showing mRNA–miRNA–lncRNA interactions. ceRNA, competing endogenous RNA; miRNA, microRNA; lncRNA, long non-coding RNA.

Drug association analysis based on the DSigDB resource identified several compounds significantly linked to the six key genes, including zinc sulfate, capsaicin, vorinostat, and dronabinol, as shown in **Figure 7A**. These drug gene associations suggest potential relevance to molecular pathways involved in MASH. To explore regulatory relationships at the post transcriptional level, a competing endogenous RNA network was assembled by integrating predicted interactions between messenger RNAs and microRNAs together with microRNA to long non coding RNA associations. The resulting network connected three key genes, namely *MMP9, CTSD*, and *TREM2*, with multiple regulatory microRNAs and long non coding RNAs, indicating a complex regulatory architecture in MASH, as illustrated in **Figure 7B**.

### 3.6 Immune cell landscape and gene associated infiltration patterns

**Figure 8:**
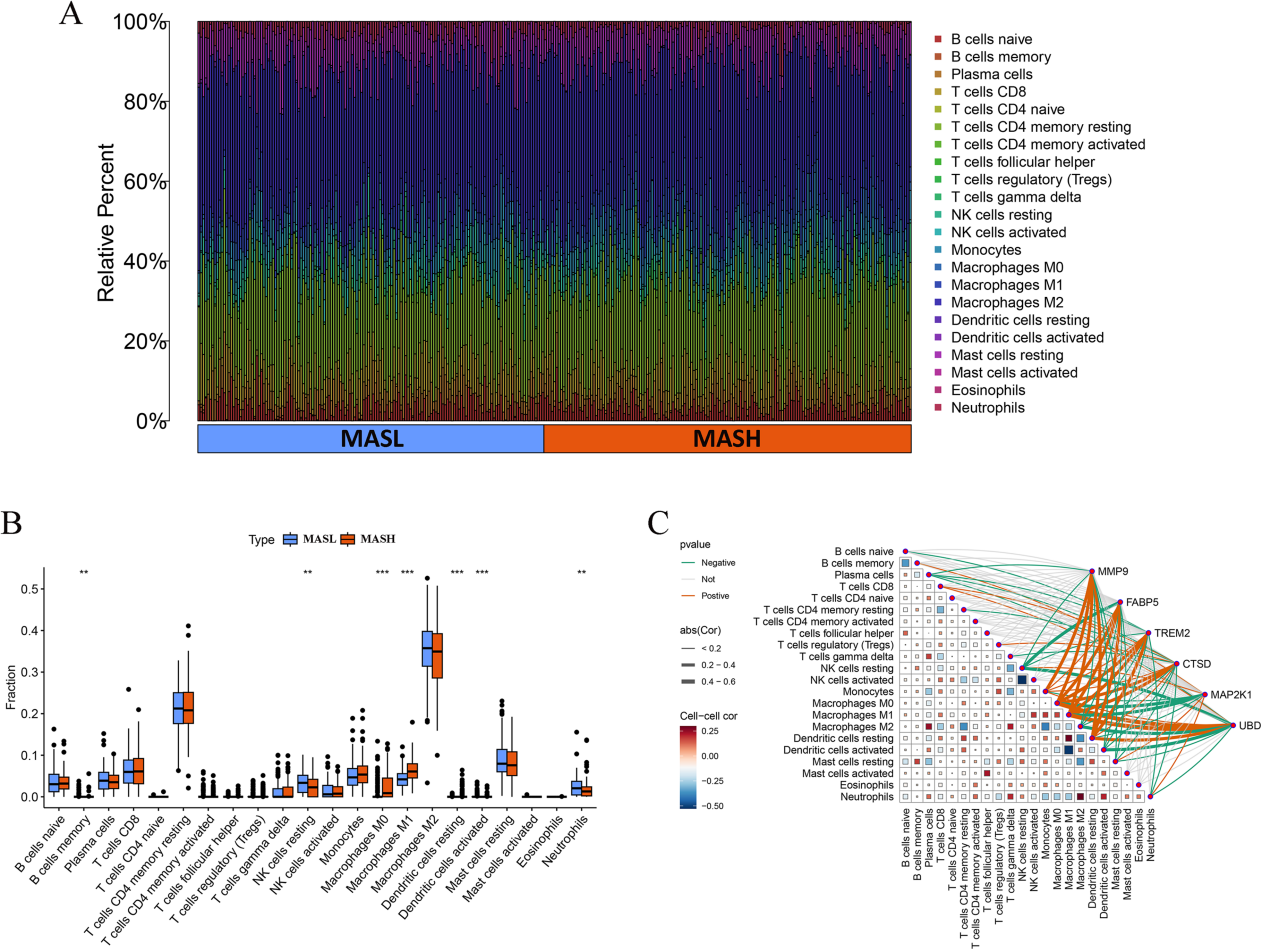
Immune infiltration analysis. **(A)** Barplot of immune cell fractions in MASL and MASH. **(B)** Boxplots showing differential infiltration of immune cell subsets. **(C)** Correlation network between key genes and immune cell infiltration. MASL, non-alcoholic fatty liver; MASH, non-alcoholic steatohepatitis.

Immune cell deconvolution using the CIBERSORT LM22 signature revealed heterogeneous yet overall comparable immune composition across samples in both MASL and MASH groups, as illustrated in **Figure 8A**. Comparative analysis between groups identified marked alterations in several immune cell subsets. Increased proportions of monocytes, M0 macrophages, M1 macrophages, activated natural killer cells, and activated dendritic cells were observed in MASH samples, whereas MASL samples exhibited higher fractions of naïve B cells, resting natural killer cells, CD8 T cells, M2 macrophages, resting dendritic cells, resting mast cells, and neutrophils, as shown in **Figure 8B**. Plasma cells, T follicular helper cells, regulatory T cells, and gamma delta T cells were present at very low levels in both groups without discernible differences. Correlation analysis linking key gene expression to immune cell fractions demonstrated that *MMP9, FABP5, TREM2, CTSD*, and *UBD* were positively associated with myeloid and activated immune compartments, particularly monocytes, M0 and M1 macrophages, and activated dendritic cells, while showing negative associations with resting natural killer cells and resting mast cells. In contrast, *MAP2K1* displayed predominantly inverse correlations with monocytes, macrophages, and activated dendritic cells. Relationships among immune cell subsets defined by the LM22 signature are summarized in the cell to cell correlation heatmap presented in **Figure 8C**.

### 3.7 Single cell transcriptomic analysis reveals cell type specific expression patterns of key genes

**Figure 9:**
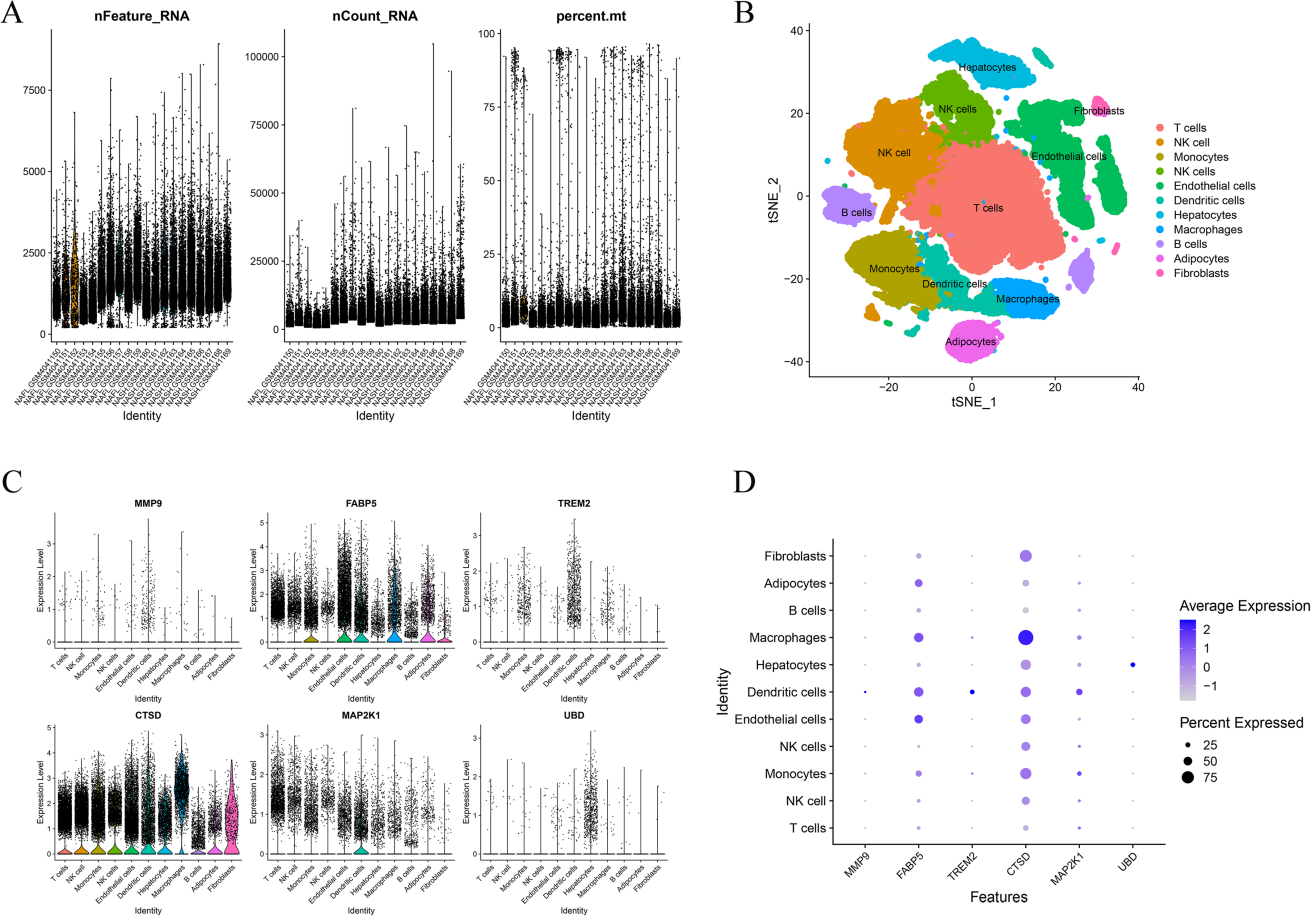
scRNA-seq overview of QC, clustering and global key-gene distribution. **(A)** QC violin plots for nFeature_RNA, nCount_RNA, percent.mt. **(B)** t-SNE colored by annotated cell types. **(C)** Violin plots of six key genes across clusters. **(D)** Dot plot showing average expression and detection rate per cell type. QC, quality control; nFeature_RNA, number of detected genes per cell; nCount_RNA, total RNA counts per cell; percent.mt, percentage of mitochondrial gene transcripts; t-SNE, t-distributed stochastic neighbor embedding.

**Figure 10:**
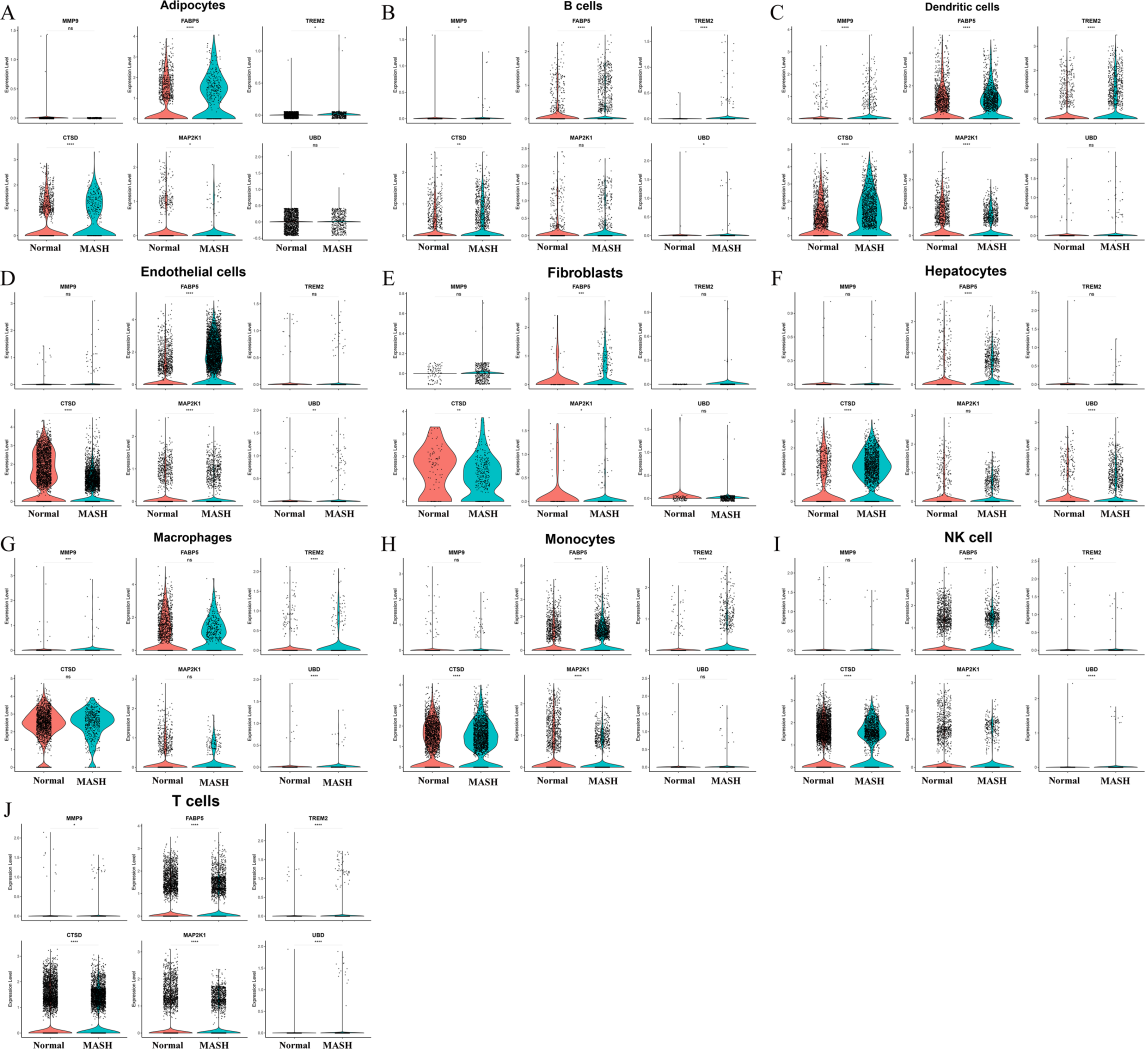
scRNA-seq validation by cell type. **(A–J)** Within each annotated cell type (Adipocytes, B cells, Dendritic cells, Endothelial cells, Fibroblasts, Hepatocytes, Macrophages, Monocytes, NK cell, T cells), violin plots compare normal vs MASH expression of the six key genes; asterisks denote Wilcoxon test significance. MASH, non-alcoholic steatohepatitis; NK cell, natural killer cell.

Single cell transcriptomic analysis of the GSE136103 dataset was performed following quality control procedures that evaluated detected gene numbers, total transcript counts, and mitochondrial transcript proportions, with these metrics summarized in violin plots shown in **Figure 9A**. Dimensionality reduction using t distributed stochastic neighbor embedding resolved major cellular populations, including T cells, natural killer cells, monocytes, macrophages, B cells, dendritic cells, endothelial cells, hepatocytes, adipocytes, and fibroblasts, as illustrated in **Figure 9B**. Violin plots across clusters together with a dot plot provided an overview of expression levels and detection frequencies of the six key genes across annotated cell types, highlighting their cellular distribution patterns in **Figure 9C** and **Figure 9D**. Single-cell RNA sequencing analysis based on the GSE136103 dataset (derived from normal and cirrhotic liver tissues) was used to characterize the cell-type-specific expression patterns of the identified key genes across major hepatic cell populations. As shown in **Figure 10**, *MMP9, FABP5, TREM2, CTSD, UBD*, and *MAP2K1* exhibited distinct expression distributions across different cell lineages. *MMP9, TREM2*, and *CTSD* were mainly expressed in myeloid cell populations, including monocytes and macrophages, indicating their association with immune-related processes. FABP5 showed expression across multiple cell types, including endothelial cells and hepatocytes, suggesting a role in metabolic regulation. *UBD* expression was relatively low and detected in a subset of immune cells. *MAP2K1* displayed a broad expression pattern across different cell types, consistent with its involvement in intracellular signaling. In dendritic cells, as well as in endothelial cells and hepatocytes, *CTSD* and *FABP5* were observed with relatively higher expression levels, while *MMP9* and *TREM2* remained primarily restricted to immune cell populations. Fibroblasts and adipocytes showed generally lower expression levels of most key genes. B cells, natural killer cells, and T cells exhibited overall modest expression across the selected genes. These results describe the cellular distribution of the identified genes within the hepatic microenvironment and provide complementary information for understanding their potential functional roles.

### 3.8 Validation of key gene expression in clinical tissue samples

**Figure 11:**
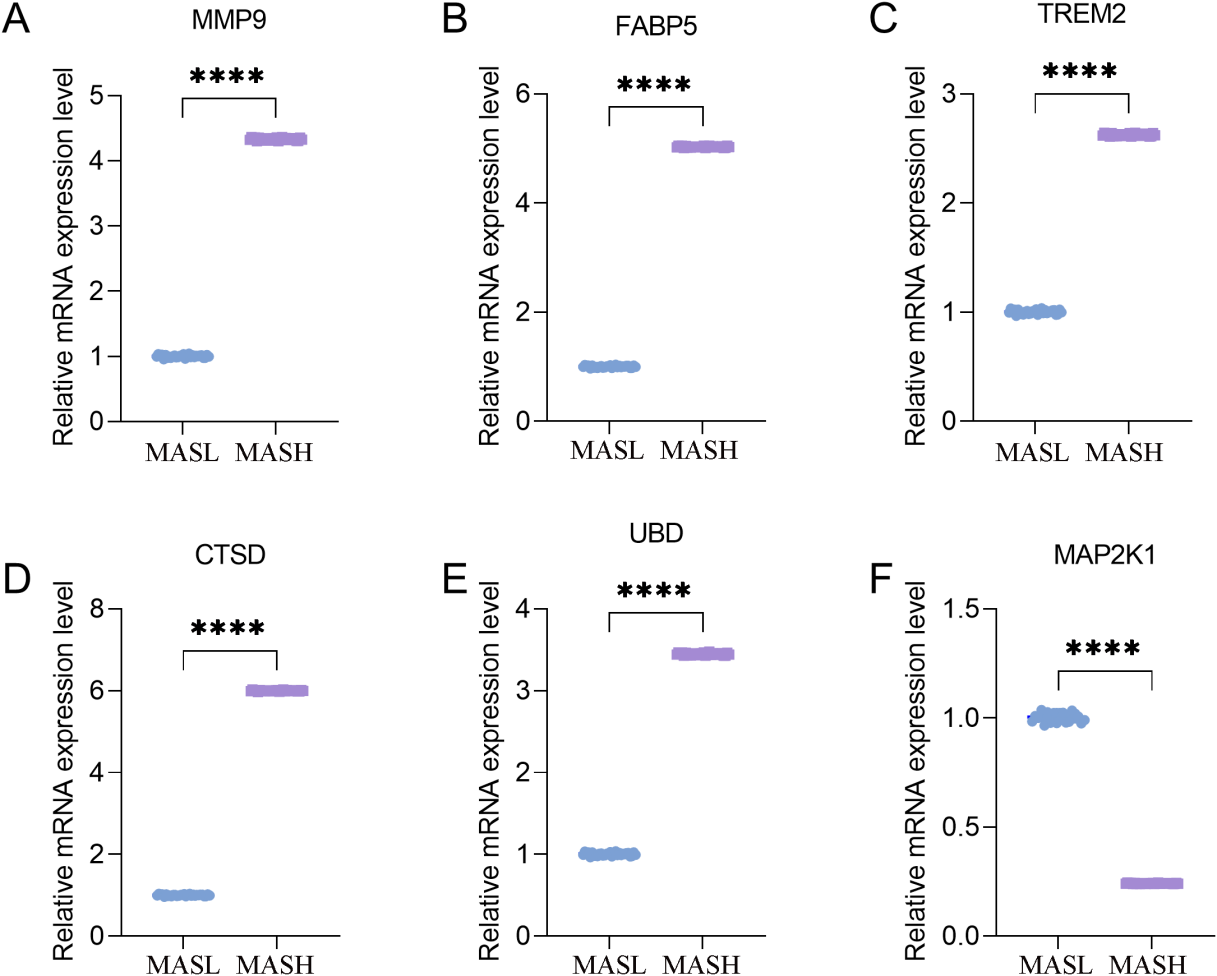
Differential mRNA expression of target genes in clinical samples **(A)***MMP9*, **(B)***FABP5*, **(C)***TREM2*, **(D)***CTSD*, **(E)***UBD*, **(F)***MAP2K1*. *MMP9*, matrix metalloproteinase 9; *FABP5*, fatty acid–binding protein 5; *TREM2*, triggering receptor expressed on myeloid cells 2; *CTSD*, cathepsin D; *UBD*, ubiquitin D; *MAP2K1*, mitogen-activated protein kinase kinase 1. **** P < 0.0001.

To experimentally validate the diagnostic genes identified through bioinformatic analyses, quantitative real time PCR was performed using sixty pathological liver tissue samples obtained from Baise People’s Hospital. A total of 60 liver tissue samples were included for qPCR validation, comprising 30 MASL and 30 MASH cases confirmed by histopathological evaluation. The mean age was 46.77 ± 9.15 years in the MASL group and 48.97 ± 12.35 years in the MASH group, with an equal sex distribution in both groups. Body mass index was markedly higher in patients with MASH (31.26 ± 2.06 kg/m²) compared with MASL (24.92 ± 1.63 kg/m²). As expected, MASH samples exhibited substantially elevated NAS scores (5.50 vs 1.57) and more advanced fibrosis stages (2.50 vs 0.37), confirming clear histopathological differences between the two groups. Detailed clinical characteristics are provided in **Supplementary Table 3**. Relative transcript levels of the six key genes, including MMP9, FABP5, TREM2, CTSD, UBD, and MAP2K1, were measured in MASL and MASH tissues, as shown in **Figure 11**. Statistically significant differences in expression were observed for all six genes between the two groups. MMP9, FABP5, TREM2, CTSD, and UBD displayed markedly higher expression in MASH tissues compared with MASL tissues, whereas MAP2K1 showed a pronounced reduction in MASH. The observed expression trends were consistent with results obtained from transcriptomic analyses, providing independent experimental support for the robustness of the screening strategy. Taken together, these findings demonstrate that the six genes exhibit distinct transcriptional patterns during progression from MASL to MASH and further support their potential utility as molecular biomarkers of disease progression.

## 4 Discussion

Early discrimination between MASL and MASH has long lacked reliable noninvasive approaches. Current clinical screening relies largely on serum biochemical parameters and composite risk scores, including liver enzymes and body mass index, yet these conventional indicators show limited sensitivity and specificity for distinguishing simple steatosis from steatohepatitis. Circulating cytokeratin 18 fragments, one of the most extensively studied biomarkers, demonstrate a sensitivity of only about 58 percent with an AUC of approximately 0.65, which is insufficient for standalone diagnosis (16). This diagnostic gap highlights the need for more informative molecular signatures. By integrating multiple public liver transcriptomic cohorts and combining weighted gene co expression network analysis, diverse machine learning algorithms, and an artificial neural network framework, we constructed a predictive model that substantially improved discrimination between MASL and MASH. The incorporation of single cell RNA sequencing analysis together with qPCR validation in clinical liver specimens further strengthened the reliability and biological interpretability of the model, offering a framework for early differentiation and for exploring potential preventive and therapeutic targets. The artificial neural network model achieved robust performance in an independent validation cohort, with an AUC of 0.893 (95 percent CI 0.854 to 0.925). For comparison, the 19 gene signature proposed by Hasin Brumshtein and colleagues yielded an AUC of 0.79 in its validation dataset (3), while serum cytokeratin 18 fragments exhibited limited sensitivity and specificity of approximately 58 percent and 68 percent, respectively (16). These differences suggest that a multigene tissue based model may capture disease specific molecular alterations more comprehensively than single biomarkers. Machine learning approaches are particularly suited to integrate latent patterns across multiple genes, thereby enhancing robustness in subtype classification. In our clinical cohort, qPCR confirmed differential expression of several model genes between MASL and MASH tissues, reinforcing their diagnostic potential.

Among the six key genes identified, *MMP9, FABP5, TREM2, CTSD*, and *UBD* were significantly upregulated in MASH, whereas *MAP2K1* was downregulated. Single cell transcriptomic mapping localized this expression pattern predominantly to immune related populations, including monocyte derived macrophages and dendritic cells, implicating these genes in immune microenvironment remodeling and metabolic dysregulation during disease progression. *MMP9*, a matrix metalloproteinase involved in extracellular matrix degradation and tissue remodeling, has been repeatedly associated with inflammatory driven structural changes and monocyte recruitment in MASH and liver fibrosis (17–19). *FABP5* plays a central role in fatty acid uptake and intracellular trafficking; its upregulation in obesity and fatty liver conditions aligns with our observation in MASH and supports its contribution to lipid metabolic imbalance (20). *TREM2* marks lipid associated macrophages that expand under metabolic stress, participating in lipid handling, inflammatory modulation, and fibrotic plasticity. Increased *TREM2* expression in MASH reflects macrophage subset reprogramming within the inflamed liver (21, 22). *CTSD*, a lysosomal protease, has been implicated in lipid metabolism and inflammatory signaling in both experimental and clinical settings (23). *UBD*, also known as *FAT10*, is a ubiquitin like modifier inducible by proinflammatory cytokines such as TNFα and IFNγ (24); its activation in MASL and MASH has been linked to modulation of metabolic regulators including PPARα (25). In contrast, *MAP2K1*, encoding MEK1, was downregulated. In the context of metabolic disorders, the role of MAPK signaling appears to be highly context-dependent. Under conditions of chronic lipid accumulation and inflammatory stress, sustained pathway activation may contribute to hepatocellular injury and inflammatory amplification, whereas insufficient signaling may impair adaptive cellular responses. The observed downregulation of *MAP2K1* in MASH may therefore reflect disrupted signaling balance, potentially affecting hepatocyte stress adaptation as well as immune cell activation within the hepatic microenvironment. These findings suggest a complex regulatory role for *MAP2K1* in disease progression, although its precise functional contribution requires further experimental validation (26). Whether this downregulation confers protection or promotes disease progression warrants further functional exploration. The concordance between bulk expression patterns and single cell localization enhances the biological plausibility of these genes as model components and suggests that targeting pathways related to MMP9 mediated matrix remodeling, TREM2 positive macrophage function, or CTSD and UBD associated metabolic inflammatory axes may hold therapeutic promise.

Immune infiltration analysis revealed pronounced differences in hepatic immune composition between MASL and MASH. MASH tissues displayed increased proportions of unpolarized M0 and classically activated M1 macrophages, accompanied by abundant infiltration of activated dendritic cells. These findings are consistent with reports describing Kupffer cell polarization toward a proinflammatory phenotype and rapid recruitment of intrahepatic dendritic cells during steatohepatitis (27). Tissue level qPCR validation further substantiated gene expression differences between disease stages. Although circulating expression was not assessed, the high tissue specificity observed here provides a foundation for developing future noninvasive biomarkers. To explore upstream regulatory mechanisms, we constructed a competing endogenous RNA network based on target predictions from miRanda, miRDB, TargetScan, and miRWalk. The analysis suggested that several long noncoding RNAs may competitively bind shared microRNAs, thereby indirectly modulating expression of key genes. Network visualization indicated that *TREM2*, *CTSD*, and *MMP9* occupied highly connected downstream positions, suggesting central regulatory roles. Although specific axes were not experimentally validated, convergence across multiple prediction platforms supports the stability of this network architecture and points to multilayered post transcriptional regulation influencing gene expression dynamics. Drug enrichment analysis using DSigDB identified significant associations between the key genes and several small molecules, including histone deacetylase inhibitors such as vorinostat, antioxidant compounds, and immunomodulatory agents (28, 29). Histone deacetylase inhibitors have demonstrated therapeutic effects in metabolic disease models through modulation of inflammatory and fibrotic gene programs (30, 31), and their potential links to *TREM2*, *CTSD*, and *MMP9* raise the possibility that epigenetic regulation may intersect with ceRNA mediated control mechanisms (32, 33). Consistently, single cell RNA sequencing confirmed enrichment of these genes in monocyte derived macrophages and dendritic cell subsets, paralleling recent descriptions of *TREM2* positive lipid associated macrophages in steatohepatitis. Although the single-cell analysis in this study primarily focused on cell-type-specific expression patterns based on a dataset derived from normal and cirrhotic liver tissues, the observed distribution of key genes suggests potential interactions among immune cell populations. Monocytes, macrophages, and dendritic cells showed consistent expression of several key genes, indicating a potential role in shaping the hepatic immune microenvironment. The upregulation of genes such as *TREM2*, *MMP9*, and *CTSD* within myeloid cells may reflect enhanced inflammatory activation and tissue remodeling, whereas the downregulation of *MAP2K1* may indicate altered intracellular signaling. Further studies incorporating cell-cell communication and trajectory analyses may help to better define these interactions and dynamic cellular processes.

Several limitations merit consideration. The analyses were confined to liver tissue samples, and independent validation in blood or other noninvasive specimens was not performed, leaving external generalizability to be determined. In addition, the validation of the model was based on retrospective public datasets. Although external validation was performed, further confirmation in prospective, multicenter cohorts is required. Future studies using more accessible sample types, such as peripheral blood or serum, may facilitate clinical translation. Future studies incorporating proteomic and metabolomic datasets, along with prospective real world cohorts, may enhance translational value. Spatial transcriptomic and tissue immunomic approaches could provide high resolution mapping of cellular interactions within the hepatic niche. Spatial analyses in healthy and fibrotic livers have already revealed distinct patterns of cellular remodeling (34), suggesting that similar strategies may clarify the microenvironmental architecture of MASH. Integrating circulating microbiome profiles, serum microRNAs, and other blood based molecular markers may ultimately enable development of practical screening tools. With further optimization, the model established here may provide a foundation for future development of clinically applicable assays or computational tools. However, further validation in non-invasive samples and prospective cohorts is required before implementation in routine clinical practice. However, its direct clinical applicability remains limited, as the current model is based on liver tissue samples and requires invasive procedures.

## 5 Conclusion

In conclusion, this study establishes a robust and biologically interpretable framework for distinguishing MASL from MASH through integrated transcriptomic and single cell analyses combined with machine learning modeling. By harmonizing multicenter liver tissue datasets and applying weighted gene co expression network analysis together with complementary machine learning strategies, we identified six key genes that capture core features of metabolic dysregulation and immune remodeling during disease progression. The artificial neural network model derived from these genes demonstrated strong diagnostic performance in independent validation, showed improved diagnostic performance compared with previously reported gene signatures in independent validation, although differences in model design and data processing should be considered when interpreting such comparisons. Beyond diagnostic accuracy, the convergence of bulk transcriptomic patterns, single cell localization, immune infiltration analysis, and clinical qPCR validation strengthens the biological plausibility of the model. The identified genes are predominantly enriched in monocyte derived macrophages and dendritic cells, underscoring the central role of immune microenvironment reprogramming in the transition from simple steatosis to steatohepatitis. Regulatory network analysis further suggests multilayered post transcriptional control and potential epigenetic modulation, providing mechanistic clues that extend beyond mere classification. Taken together, these findings indicate that a multigene, tissue based molecular signature can improve early identification of high risk patients and offer insights into actionable pathways involved in MASL progression. With additional validation in prospective and noninvasive cohorts, this strategy may contribute to the development of clinically applicable tools for risk stratification, early diagnosis, and targeted intervention, ultimately reducing dependence on liver biopsy and improving outcomes in patients with MASLD spectrum disorders.

## Data Availability

The original contributions presented in the study are included in the article/**Supplementary Material**, further inquiries can be directed to the corresponding authors.
